# Clinical assessment of a new wearable tool for continuous and objective recording of motor fluctuations and ON/OFF states in patients with Parkinson’s disease

**DOI:** 10.1371/journal.pone.0287139

**Published:** 2023-10-05

**Authors:** Luigi Battista, Miriam Casali, Livia Brusa, Fabiana Giada Radicati, Fabrizio Stocchi

**Affiliations:** 1 R&D Department, Biomedical Lab SRL, Potenza, Italy; 2 Department of Neurology, Institute of Research and Medical Care IRCCS San Raffaele, Rome, Italy; 3 Department of Neurology, Ospedale S. Eugenio, Rome, Italy; Clinical Investigation Center, TUNISIA

## Abstract

Clinical rating scales typically includes subjective evaluations, and their time-limited duration may fail to capture daily fluctuations in motor symptoms resulting from Parkinson’s disease (PD). Recently, a new tool (i.e. the PD-Watch) has been proposed for the objective and continuous assessment of PD motor manifestations based on evaluating frequency data from a wrist-worn tri-axial accelerometer and identifying specific movement patterns typically associated with disorders. This reduces the probability of confusing physiological or pathological movements occurring at the same frequency. In this work, we present a new method for assessing motor fluctuations through a wrist-worn accelerometer. We also explore the agreement between the continuous data generated by the proposed method and data reported in the patient diaries. In this study, twelve PD patients were recruited with an overall recording duration of 528 hours. Results of this preliminary study show that the proposed tool has suitable and adequate performances for analysing the motor signs of PD patients, and the estimated sensitivity, specificity, and accuracy of the tool are 85%, 94%, and 91%, respectively.

## I. Introduction

The current gold standard for the evaluation of motor performances and impairments of Parkinson’s disease (PD) patients include the execution of neurological examinations, the use of the information provided by the patient, such as their motor symptoms diary, and rating scales, like the Unified Parkinson’s Disease Rating Scale (UPDRS) [1–5]. Nevertheless, the time-limited duration of the above-mentioned clinical examinations may typically fail to capture daily fluctuations in PD motor symptoms [6, 7]. In order to reduce these shortcomings, various technology-based objective measures (TOMs) have been proposed [8–20]. Some solutions are based on wearable inertial measuring units and signal processing to evaluate the frequency content in the range in which motor symptoms due to PD typically occur (i.e. mainly up to about 12 Hz) [2, 21]. However, most typical habitual motor activities performed by patients may have a power spectrum (e.g. up to 20 Hz [22]) that overlaps with the range in which regular movements typically occur. As a consequence, solely evaluating the frequency content does not usually offer a reasonable distinction between movement disorders and normal daily motor activity.

In order to improve this, a recent tool (i.e. "PD-Watch") has been proposed for the continuous assessment of tremor, bradykinesia, and dyskinesia through a wrist-worn accelerometer [23, 24]; the PD-Watch evaluates the frequency data content coming from multi-axial sensors and identifies specific movement patterns that motor symptoms are typically associated with [23] (e.g. PD hand tremors usually occur between 3 Hz and 7 Hz with a supination–pronation characteristic [2]). With reference to PD-induced hand tremor detection, this tool checks if the movement frequency falls within the above-quoted typical range and if it has a supination–pronation pattern [23]. This may reduce the probability of being misled by other physiological or pathological movements occurring at the same frequency as PD tremors but with a movement pattern that differs from the characteristic supination–pronation motion.

In this work, we present the PD-Watch method for assessing ON and OFF motor states through a wrist-worn accelerometer and we explore the agreement between the continuous data generated by the proposed method and data reported in the patient diaries.

## II. Materials and methods

**Fig 1:**
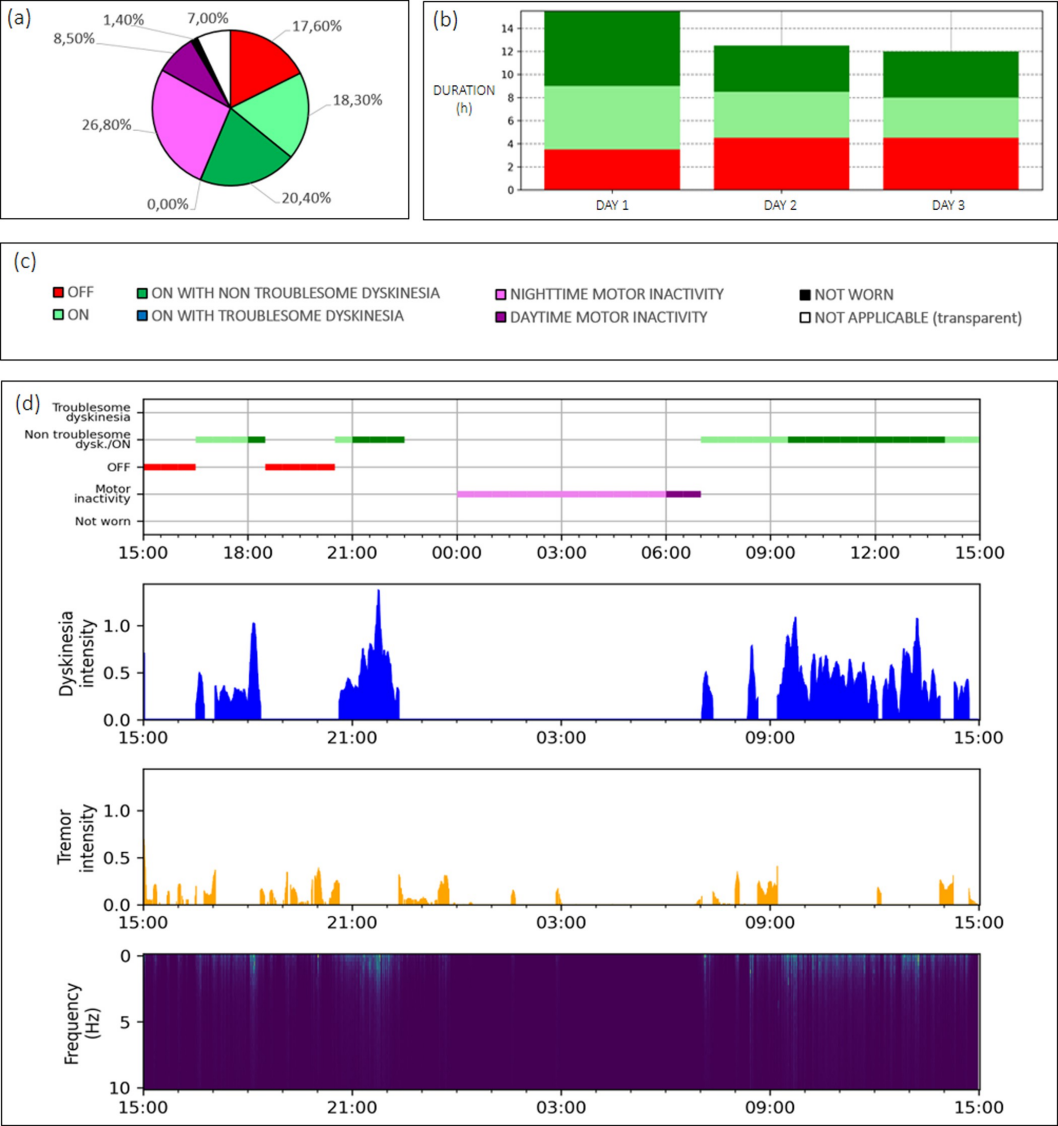
Data obtained through the PD-Watch tool for a 72-h acquisition of a PD patient with motor fluctuations. (a) Pie chart on the percentage duration of the recorded motor states. (b) Daily histograms with motor state duration. (c) Color legend. (d) Temporal patterns provided by the PD-Watch for a 24-h sequence of the whole 72-h acquisition. First row: temporal patterns of motor states; second row: temporal pattern of dyskinesia intensity; third row: the temporal pattern of tremor intensity; fourth row: a spectrogram of the 24-h recording.

In this study, twelve PD patients were recruited (6 males and 6 females, mean age 65, age from 59 to 71), nine patients from the Department of Neurology, Institute of Research and Medical Care, IRCCS San Raffaele, Rome, Italy, and three patients from the Neurology Unit of the Hospital “Sant’Eugenio” of Rome, Italy. Patients provided written informed consent prior to participation in the study; the study protocol was reviewed and approved by the Ethics Committee of Basilicata "CEUR", Italy, before the study began. The UPRDS scoring was performed by two examiners and with patients under medication; the final results of UPRDS scoring were obtained by calculating the mean value of the two different ratings. After neurological examination, the continuous recording of movements began; patients were asked to wear the portable system on their most affected wrist for continuous recording of motor activity in each patient’s normal environment. This wrist-worn device comprised a tri-axial accelerometer, a battery, memory support, a near-body thermistor, a lux meter, and a microcontroller unit; it allows to collect data with a frequency of 50 Hz per channel and up to fifteen consecutive days. The recording period was set from twenty-four to seventy-two consecutive hours, based on the patients’ conditions and/or the caregivers availability, and at the end of this acquisition period, recorded data were processed with the PD-Watch algorithm. Fig 1 summarizes the main information provided by the PD-Watch for a PD patient with motor fluctuations; raw-data provided by the wearable tool are reported in S1 Fig of S1 File.

Before the beginning of the recording session, patients were instructed by the clinical staff to fill out the diary and distinguish between the four motor states available in the motor symptoms diary (Asleep, OFF, ON/ON with non-troublesome dyskinesia, ON with troublesome dyskinesia), according to the findings of a previous study [25]. At the end of the acquisition period, the clinical staff discussed the completed motor symptoms diaries with the patients. The results provided by the PD-Watch were compared to data reported in the patient symptoms diaries by considering thirty-minute intervals. All relevant data are within the manuscript and S1 File.

### Motor signs assessment

The method for detecting motor signs was already reported [23] and is summarised below. In the pre-processing phase, data of the entire acquisition were divided into equal time sequences; for each time sequence, the Power Spectral Density (PSD) was computed for each axis of the acceleration signal, and the root mean square value of the three axes.

A tremor is determined for the time sequences where a pronation-supination movement determining a maximum value in the frequency range between 3 Hz and 7 Hz is observed. The identification of pronation-supination movement is based on the inter-axes comparison of the frequency contents detected on each axis of the tri-axial accelerometer [23]. Finally, the tremor intensity may be provided from 0 to 4, just like MDS-UPDRS scores, by correlating the numerical values computed from the integration of PSD between 3 Hz and 7 Hz [24].

Dyskinesia is determined if a combination of events occurs. Firstly, the dyskinesia identification is based on the calculation of the PSD integral between 1 Hz and 3 Hz and between 3 Hz and 8 Hz for each sequence and in the observation of a wider temporal window (e.g. 30 minutes); dyskinesia is detected if such calculated values are above a certain threshold and if this condition occurs for most of the time sequences contained in that temporal window. This analysis prevails on possible isolated tremor events and considers that patients with dyskinesia are infrequently motionless, and their movements are typically characterised by higher spectral energy and higher intensity than a typical person [23]. Finally, the dyskinesia intensity may be provided from 0 to 4, just like MDS-UPDRS and AIMS (Abnormal Involuntary Movement Scale) scores, by correlating the numerical values computed from the integration of PSD between 1 Hz and 8 Hz. The above quoted thresholds on dyskinesia have been defined by considering PD patients with clinically diagnosed dyskinesia.

Finally, in the authors’ opinion, the detection of bradykinesia with a single accelerometer is still challenging because it is taxing to reliably distinguish voluntary slow movements between slowness due to PD pathological conditions. As reported in the section “Discussion”, in order to reduce this, the bradykinesia is detected only if the patient is in a standing position or in a non-sedentary condition, when the probability of detection of the slowness due to pathological condition and distinguish it from voluntary slow movements is likely higher, because most of the daily voluntary slow movements and rest/sedentary periods typically occur when the patient is not standing; this is also consistent with the circumstance that motor tasks for the detection of bradykinesia are also carried out with patients in a standing position [26]. Therefore, bradykinesia is determined if a combination of events occurs. Firstly, the bradykinesia identification is based on the detection of body position and the calculation of the PSD integral between 0.5 Hz and 3 Hz; bradykinesia is detected if the patient is standing and if the above-quoted calculated values are within two thresholds, the first one to exclude motionless conditions (e.g. voluntary rest and sedentary periods) and the second one to exclude physiological and pathological movements that are not slow. Finally, the bradykinesia intensity may be provided from 0 to 4, just like MDS-UPDRS, by correlating the inverse values of the above-quoted numerical measures computed from the integration of PSD between 0.5 Hz and 3 Hz.

The above quoted thresholds have been defined by considering patients with clinically diagnosed PD.

### ON/OFF states detection

For PD patients with motor fluctuations, the PD-Watch method provides the ON/OFF motor state detection in thirty-minute time intervals by considering the information on tremor, bradykinesia, and dyskinesia detected in that time period (Fig 1); for PD patients without motor fluctuation, the PD-Watch reports information on tremor and bradykinesia (S2 Fig of S1 File). The description of each motor state detected in patients with motor fluctuations is reported below:

- OFF state is determined when bradykinesia or the combination of bradykinesia and tremor is predominant with respect to the other motor manifestations in that period;
- ON state is determined when motor activity is detected during the analysed time period, and no relevant/disabling bradykinesia is detected, nor relevant/disabling tremor and possible detected dyskinesias are not impactful;
- ON WITH NON-TROUBLESOME DYSKINESIA state is determined when dyskinesias are observed for the majority of the analysed time and with a maximum dyskinesia intensity score greater than 0.5 and lower than 2.5;
- ON WITH TROUBLESOME DYSKINESIA state is determined when dyskinesias are observed for the overwhelming majority of the analysed time and with a maximum dyskinesia intensity score greater than 2.5;
- DAYTIME MOTOR INACTIVITY state is determined when the wearable system is worn in the analysed time, but reduced motor activity is detected. This concerns the time periods occurring during the daytime (e.g. 6:00 to 22:00);
- NIGHTTIME MOTOR INACTIVITY state is determined when the wearable system is worn in the analysed time, but reduced motor activity is detected. This concerns the time periods occurring during the nighttime (e.g. 22:00 to 6:00);
- NOT WORN state is determined when it is detected that the wearable system is not worn for the majority of the analysed time. The near-body thermometer and/or the accelerometer enclosed in the wearable system may be used to detect if it is worn or not;
- NOT DETECTABLE or NOT APPLICABLE state is determined when it is not possible to assign none of the above-quoted states.

### Comparison between patient diary data and the PD-Watch

The results of the ON-OFF states provided by the PD-Watch were compared to data reported in the patient symptoms diary by considering thirty-minute intervals. The comparison aims to evaluate the performances of the proposed tool and method in distinguishing the various motor states, exploring overall general performances and the performances in ON state detection and OFF state detection separately.

Therefore, for each thirty-minute interval, we consider a true positive (TP) when an OFF state was properly determined and a false positive (FP) when an OFF state was determined while the patient was not in an OFF state. Moreover, we consider a true negative (TN) when a state different from OFF state was determined while the patient was not in an OFF state and a false negative (FN) when a state different from OFF state was determined while the patient was in an OFF state. Similarly, TP, FP, TN and FN were determined for the ON states. For each thirty-minute interval where the proposed tool reported NOT WORN or NOT DETECTABLE state, the assignment of TP, FP, TN and FN was not carried out.

### Statistical analysis

The Cohen’s Kappa statistic was used to measure the agreement between motor states provided by the PD-Watch and data reported in the patient symptoms diary. The Cohen’s Kappa coefficient was obtained for each 24-h acquisition according to [27]:

$$k\;=\;\frac{2\times (TP\times TN-FN\times FP)}{\left(TP+FP)\times (FP+TN)+(TP+FN)\times (FN+TN\right)}$$

The specificity, sensitivity and accuracy were then quantified according to:

$$\;Specificity\;=\;\frac{TN}{TN+FP}$$

$$\;Sensitivity\;=\;\frac{TP}{TP+FN}$$

$$Accuracy\;=\;\frac{TP+TN}{TP+TN+FP+FN}$$

## III. Results

A summary of the data reported in the patient diaries and the data provided by the PD-Watch found for each 24-h acquisition period, for a total of five hundred and twenty-eight recording hours, are shown in Tables 1 and 2 and S1 Table of S1 File. The average worn time was of 2.2 days per patients.

**Table 1:** A comparison between the data contained in the patient diaries and data provided in the PD-Watch report for each 24-h acquisition period. The overall recording duration of 528 hours with twelve PD patients is divided in no. 22 acquisition periods, with a 24-h duration for each acquisition period. The symbol “#” is used to identify each 24-h acquisition period.

#	PATIENT DIARY				PD-WATCH REPORT					
	OFF (h)	ON/ON with non-troublesome dyskinesia (h)	ON with troublesome dyskinesia (h)	Sleeping/Motor Inactivity (h)	OFF (h)	ON/ON with non-troublesome dyskinesia (h)	ON with troublesome dyskinesia (h)	Motor Inactivity (h)	N. A. (h)	Worn Period (% of the day)
1	4	11,5	0	5,5	3	13,5	0	4,5	1	95%
2	2,5	11,5	0	7,5	4	10	0	7,5	1,5	100%
3	2	13	0	1,5	3	12	0	1,5	1	79%
4	2	8,5	0	8	1	9,5	0	8	0	79%
5	1,5	10	0	10	5	7	0	9,5	0,5	100%
6	2,5	10,5	0	7,5	5,5	6,5	0	8,5	1,5	100%
7	1,5	4,5	0	7	1	5	0	7	0,5	60%
8	3	9	1	6,5	3	7	1	8,5	3,5	99%
9	1	15	0	4,5	1,5	12,5	0,5	6	1,5	100%
10	1	7,5	0	6,5	0,5	8	0	6,5	1,5	76%
11	3,5	10,5	0,5	8,5	6	8	0,5	8,5	1	100%
12	2,5	8,5	0	7	1,5	9,5	0	7	2	85%
13	8	4	1	10	8	3	1	11	0,5	100%
14	3,5	11	0	8,5	2	11,5	0	9,5	0	100%
15	7,5	6	0	7,5	6	5,5	0	9,5	2	100%
16	1,5	16	0	5	2,5	15	0	5	1	100%
17	2,5	12	0	9,5	3,5	12	0	7	1,5	100%
18	4,5	9,5	0	10	4,5	8	0	9,5	2	100%
19	2,5	11,5	0	10	4,5	7,5	0	8,5	1,5	92%
20	2	14	0	8	2,5	13	0	6,5	2	100%
21	3,5	13,5	0	7	4,5	13	0	5,5	1	100%
22	4,5	11	0	8,5	4	10,5	0	6,5	1,5	94%

**Table 2:** Outcome of the comparison between the data contained in the patient diaries and data provided in the PD-Watch report for each 24-h acquisition period. The overall recording duration of 528 hours with twelve PD patients is divided in no. 22 acquisition periods, with a 24-h duration for each acquisition period. The symbol “#” is used to identify each 24-h acquisition period.

#	COMPARISON OUTCOME AND PERFORMANCES						
	True Negative	True Positive	False Negative	False Positive	Sensitivity	Specificity	Accuracy
1	49	29	2	4	0,94	0,92	0,93
2	54	24	3	3	0,89	0,95	0,93
3	32	26	4	4	0,87	0,89	0,88
4	51	19	2	2	0,90	0,96	0,95
5	56	17	7	6	0,71	0,90	0,85
6	50	16	8	8	0,67	0,86	0,80
7	39	11	1	1	0,92	0,98	0,96
8	46	20	6	2	0,77	0,96	0,89
9	48	27	5	2	0,84	0,96	0,91
10	46	16	1	1	0,94	0,98	0,97
11	55	21	7	7	0,75	0,89	0,84
12	48	20	2	2	0,91	0,96	0,94
13	64	22	4	2	0,85	0,97	0,93
14	61	26	3	2	0,9	0,97	0,95
15	55	23	4	2	0,85	0,96	0,93
16	53	33	2	2	0,85	0,96	0,93
17	58	27	4	1	0,87	0,98	0,94
18	59	20	3	4	0,87	0,94	0,92
19	56	19	3	6	0,86	0,90	0,89
20	57	28	3	2	0,90	0,97	0,94
21	48	27	6	5	0,82	0,91	0,87
22	52	25	3	4	0,89	0,93	0,92
**MEAN VALUE**					**0,85**	**0,94**	**0,91**

Furthermore, Tables 1 and 2 and S1 Table of S1 File illustrate the outcome of the comparison between data contained in the patient diaries and in the PD-Watch reports; in particular, a detailed analysis of the TP, FP, TN and FN found for each 24-h acquisition period is reported together with the associated values of specificity, sensitivity, and accuracy and the outcome on the Cohen’s Kappa statistic. Table 1 also shows the percent of the time of day with the wearable system properly worn, as detected by the PD-Watch. Some 24-h acquisition periods were excluded from the analysis and are not reported in Table 1; some patients reported they forgot to wear the portable system or to fill out the diary at various times of the day; another patient required hospitalisation, so that acquisition was excluded because the patient was not in his typical home/daily environment; finally, another acquisition was excluded because the recruited patient was not affected by motor fluctuation (results on tremor and bradykinesia are reported in S2 Fig of S1 File). The mean Cohen’s Kappa coefficient *k* obtained for the 24-h acquisition periods is 0.80 with a standard deviation of 0.10.

The average overall sensitivity, specificity, and accuracy detected for the 24-h acquisition periods are 85%, 94%, and 91%, respectively, as detailed in Table 2 and in S1 Table of S1 File. These mean values are determined by considering each 24-h acquisition period; in the best acquisition, the specificity, sensitivity, and accuracy are 94%, 98%, and 97%, respectively, whereas in the worst acquisition, the specificity, sensitivity, and accuracy are 67%, 86%, and 80%, respectively. Finally, considering the OFF and ON detection separately, the average specificity, sensitivity, and accuracy are 92%, 92%, and 92% for ON detection and 68%, 96%, and 91% for OFF detection.

**Fig 2:**
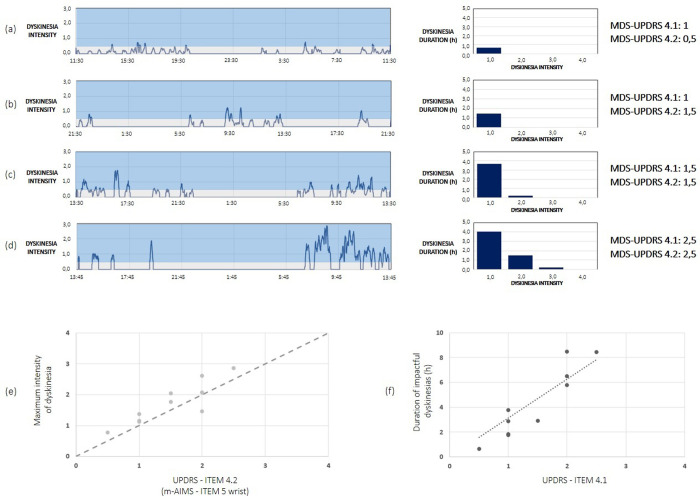
(a-d) Information on dyskinesia detected during 24-hour acquisitions for four different patients, from (a) to (d). First column: 24-h temporal pattern of dyskinesia intensity; second column: histogram of the time spent with impactful dyskinesia distributed for the different score values from 1 to 4; third column: the value of the MDS-UPDRS score for the items 4.1. and 4.2. (e-f) shows a comparison between the information on dyskinesia maximum intensity and duration detected by the tool and the MDS-UPDRS score for the items related to dyskinesia. (e) The relationship between the scores of item 4.2 of the MDS-UPDRS on the functional impact of the dyskinesias (comparable to the item 5 –wrist–of the AIMS) and the maximum intensity of the dyskinesia. (f) The relationship between the scores of item 4.1 of the MDS-UPDRS on the time/percent of the waking day spent with dyskinesia and the duration of impactful dyskinesia.

The study involved PD patients with motor fluctuations and with different stages of the disease. As an example, Fig 2A–2D shows the information on dyskinesias detected by the PD-Watch during the 24-h acquisition period for four patients with varying scores of the items 4.1. and 4.2 of the MDS-UPDRS related to the time spent with dyskinesias and the functional impact of the dyskinesias [4, 5]. In particular, the first column of the Fig 2A–2D report shows the temporal pattern of the intensity of the dyskinesia detected by the PD-Watch during the 24-h acquisition period for each patient; in the second column, the histogram summarises the time spent for each patient with dyskinesia, by subdividing the duration for each intensity score. In particular, the values of dyskinesia intensity lower than 0.5 were not computed in the whole dyskinesia duration because this condition is referred to as a low severity of dyskinesia which is likely not impactful (in the first column of Fig 2A–2D, this range appears with a grey background); for the other scores (light blue background), the intensity score of 1 refers to values from 0.5 to 1.5, the intensity score of 2 refers to values from 1.5 to 2.5, the intensity score of 3 refers to values from 2.5 to 3.5, the intensity score of 4 refers to values from 3.5 to 4. Finally, the third column reports the MDS-UPDRS score for the items 4.1. and 4.2 for each patient.

Fig 2A–2D shows that higher values of such dyskinesia items of the MDS-UPDRS correspond to higher values of the maximum intensity of the dyskinesia and higher values of dyskinesia duration. Indeed, Fig 2E shows a fair agreement between the scores of item 4.2 of the MDS-UPDRS and the maximum intensity of the dyskinesia (coefficient of determination *r*^*2*^ of the linear regression equal to 0.681). Moreover, Fig 2F shows a good agreement between the scores of item 4.1 of the MDS-UPDRS on the time/percent of the waking day spent with dyskinesia and the duration of impactful dyskinesia (intensity greater than 0.5) with a coefficient of determination *r*^*2*^ of the linear regression equal to 0.811 (we have considered a “waking day” of 16 h in the calculation of the score of the items 4.1 of the MDS-UPDRS [4, 5]).

**Fig 3:**
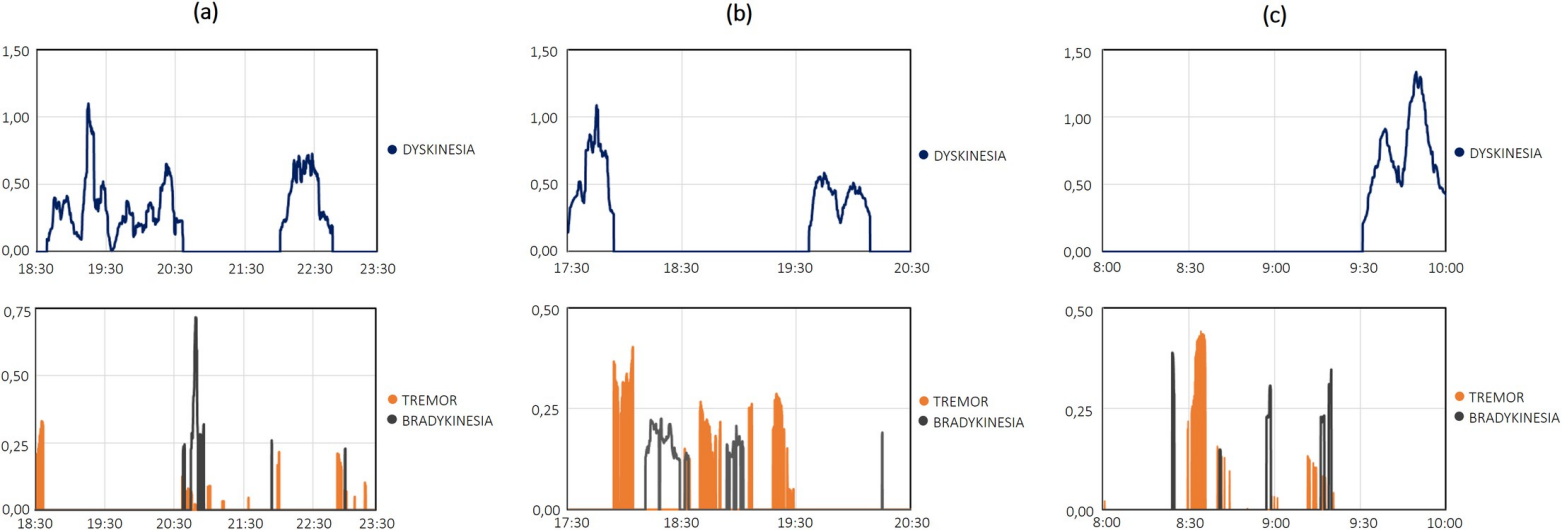
Examples of clinical application cases with temporal patterns of dyskinesia, tremor, and bradykinesia detected by the tool. (a-b) Temporal patterns related to clinical application cases concerning the wearing-off phenomenon. (c) Temporal patterns related to clinical application cases concerning the early-morning OFF period.

Finally, some clinical application cases are reported in Fig 3 and S3 Fig of S1 File. Fig 3A–3C shows the pattern of motor signs for a few hours, as detected with the PD-Watch; Fig 3A and 3B report two different examples of a PD patient experiencing a wearing-off period. Indeed, in Fig 3A, from a functional ON state with slight dyskinesias, there is the first transition to an OFF state with the presence of tremor and bradykinesia and, after about one hour and a half, a second transition to an ON state with slight dyskinesia and without relevant tremor and bradykinesia. A similar example is reported in Fig 3B, where the wearing-off duration is slightly greater than the previous example (i.e. about 1 hour and 40 minutes). Then, an example of the early-morning OFF phenomenon is shown in Fig 3C with a transition from OFF and ON state during early morning daytime.

Lastly, S3 Fig of S1 File shows the temporal patterns provided by the PD-Watch for two consecutive days of an acquisition in a PD patient starting at about 12:15; results obtained for the first day are reported in the first column of S3 Fig in S1 File, and similarly, the results of the second day are available in the second column. For each 24-h acquisition, the first row shows a graph with the temporal pattern of motor state; the second row illustrates the temporal pattern of dyskinesia intensity; the third row reports the temporal pattern of tremor and bradykinesia intensity. Finally, there is the spectrogram of the recording in the fourth row. During the discussion of the motor symptoms diary after the recording session, the patient communicated to the clinical staff he typically spent most of the daytime in an ON state, as confirmed by the results of the PD-Watch report for the first day. However, the patient also reported that, during the 24-h acquisition on the second day, he forgot to follow the treatment plan based on oral therapy. This lack of patient adherence determined the severe increase of the time spent in an OFF state during daytime with respect to the usual condition in which the patient experienced a good response to the medication. Indeed, as reported in S3 Fig of S1 File, the slight/mild dyskinesias occur from about noon to the evening during the day, with medication completely disappearing on the second day without patient adherence, whereas tremor and bradykinesia are, as a whole, higher than the previous day. Furthermore, the spectrogram shows the important difference in the presence of motion during the first day and the absence/reduction during the afternoon of the second day.

## IV. Discussion

Currently, available methods for the objective and continuous assessment of motor manifestations of PD may allow monitoring of the patient’s continuously changing motor state for the whole day. Most of these systems are based on the use of wearable magneto-inertial sensors placed at wrist or at other body positions (e.g. waist or combination of various body locations, such as wrist and ankle) [9–20, 28]. Some TOMs are based on the wearable sensors placed in body parts different from the wrist and are not able to provide information on hand tremors. Other systems detect the presence of the dyskinesia but are not able to provide information on dyskinesia severity and the algorithm makes no distinction between troublesome and non-troublesome dyskinesia, which is crucial for decision-making in the clinic. Finally, some TOMs do not provide continuous information on bradykinesia/slowness, but only provide a sample monitoring based on the evaluation of simple motor tests performed by the patients in specific time period of the day, other tools do not report the information on ON/OFF motor states.

Still, some systems do not allow a reasonable distinction between movement disorders and normal daily motor activity. Indeed, they are mainly based on spectral analysis to check if the detected signal frequency is within the range in which motor signs due to PD typically occur. But the mere evaluation of this usually does not allow an accurate distinction because the usual frequency range of physiological and pathological movements may overlap. Recently, the PD-Watch tool has been proposed based on the combination of the evaluation of frequency content of data from multi-axial sensors and of the identification of specific movement patterns with which movement disorders are typically associated; thus, potentially reducing the probability of confusion between movement disorder detection by other physiological or pathological movements occurring at the same frequency but with a different movement pattern. The information provided by the PD-Watch include tremor, bradykinesia, dyskinesia and ON/OFF motor states for 24 hours per day and up to 15 consecutive days, with the PD-Watch algorithm able to make distinction between troublesome and non-troublesome dyskinesia as reported above.

A comparison between data provided by the PD-Watch tool and information in the patient diaries is reported and discussed here. Results of such comparison show a good agreement, since the average values of the overall sensitivity, specificity, and accuracy determined for the 24-h acquisition periods are equal to 85%, 94%, and 91%, respectively. Such outcomes may be considered fair and adequate performances in the current clinical practice for motor assessment, also taking into account the very good agreement coming from Kappa statistic (average *k* value equal to 0.80).

It should be noted that the above-quoted comparison is carried out between elements that are not completely homogeneous. Indeed, the considered tool may allow for the continuous, objective, and quantitative recording of some motor aspects, but no information on non-motor aspects are provided; conversely, UPDRS/AIMS scoring is not continuous and may be subjective, whereas patient diaries are not always reliable. Therefore, possible improvements for future studies may include the continuous presence of a PD expert rater during the observation period and/or video monitoring, just like the recruitment of more PD patients.

In any case, as discussed above, this is a preliminary study carried out with a limited sample of patients and, as a consequence, results need to be extended with further studies.

## Supporting information

S1 File Supporting information reports more details on the study protocol, the inclusion criteria and the performances of the proposed tool. The Supplementary figures show some examples of data obtained through the PD-Watch tool, including an application example related to the patient adherence to the oral therapy plan.
(DOCX)
