# Supplementary material for: Clinical assessment of a new wearable tool for continuous and objective recording of motor fluctuations and ON/OFF states in patients with Parkinson’s disease
Source: PLoS One. 2023 Oct 5;18(10):e0287139. doi: 10.1371/journal.pone.0287139 (PMC10553324; doi:10.1371/journal.pone.0287139)
Supplement: S1 File — The Supplementary figures show some examples of data obtained through the PD-Watch tool, including an application example related to the patient adherence to the oral therapy plan. (DOCX) [file pone.0287139.s001.docx]

**Supporting Information file**

The interventional study (without drug) was conducted on a sample of 12 patients. The recruitment was carried out following a convenience sampling among patients from the two different medical centers (i.e. Institute of Research and Medical Care “IRCCS San Raffaele”, Rome, Italy, and Hospital “S. Eugenio”, Rome, Italy) and the following inclusion criteria were applied: a clinical diagnosis of PD; tremor; bradykinesia; motor fluctuations and/or dyskinesia; more than 50 years of age; patients able to fill out the diary and distinguish between the four motor states available in the motor symptoms diary (Asleep, OFF, ON/ON with non-troublesome dyskinesia, ON with troublesome dyskinesia) after instruction session provided by medical staff.

The recruited patients were in a stage greater than 2 of the Modified Hoehn and Yahr Scale (stage from mild/moderate involvement) and with a mild-to-moderate bradykinesia.

**Table Footnotes**

**Table S1.** A comparison between the data contained in the patient diaries and data provided in the PD-Watch report with details on OFF and ON detection. (a) Details on True Negative, True Positive, False Negative and False Positive. (b) Details on Sensitivity, Specificity and Accuracy.

(a)

| **#** |  | **COMPARISON OUTCOME AND PERFORMANCES - OFF DETECTION** | | | |  | **COMPARISON OUTCOME AND PERFORMANCES - ON DETENCTION** | | | |  | **COMPARISON OUTCOME AND PERFORMANCES - GLOBAL VALUES** | | | |
| --- | --- | --- | --- | --- | --- | --- | --- | --- | --- | --- | --- | --- | --- | --- | --- |
|  |  | **True Negative** | **True Positive** | **False Negative** | **False Positive** |  | **True Negative** | **True Positive** | **False Negative** | **False Positive** |  | **True Negative** | **True Positive** | **False Negative** | **False Positive** |
| 1 |  | 34 | 6 | 2 | 0 |  | 15 | 23 | 0 | 4 |  | 49 | 29 | 2 | 4 |
| 2 |  | 34 | 5 | 0 | 3 |  | 20 | 19 | 3 | 0 |  | 54 | 24 | 3 | 3 |
| 3 |  | 26 | 3 | 1 | 3 |  | 6 | 23 | 3 | 1 |  | 32 | 26 | 4 | 4 |
| 4 |  | 33 | 2 | 2 | 0 |  | 18 | 17 | 0 | 2 |  | 51 | 19 | 2 | 2 |
| 5 |  | 33 | 3 | 1 | 6 |  | 23 | 14 | 6 | 0 |  | 56 | 17 | 7 | 6 |
| 6 |  | 28 | 5 | 2 | 6 |  | 22 | 11 | 6 | 2 |  | 50 | 16 | 8 | 8 |
| 7 |  | 23 | 2 | 1 | 0 |  | 16 | 9 | 0 | 1 |  | 39 | 11 | 1 | 1 |
| 8 |  | 30 | 5 | 1 | 1 |  | 16 | 15 | 5 | 1 |  | 46 | 20 | 6 | 2 |
| 9 |  | 37 | 1 | 1 | 2 |  | 11 | 26 | 4 | 0 |  | 48 | 27 | 5 | 2 |
| 10 |  | 30 | 1 | 1 | 0 |  | 16 | 15 | 0 | 1 |  | 46 | 16 | 1 | 1 |
| 11 |  | 32 | 6 | 7 | 0 |  | 23 | 15 | 0 | 7 |  | 55 | 21 | 7 | 7 |
| 12 |  | 31 | 3 | 2 | 0 |  | 17 | 17 | 0 | 2 |  | 48 | 20 | 2 | 2 |
| 13 |  | 28 | 14 | 2 | 2 |  | 36 | 8 | 2 | 0 |  | 64 | 22 | 4 | 2 |
| 14 |  | 39 | 4 | 3 | 0 |  | 22 | 22 | 0 | 2 |  | 61 | 26 | 3 | 2 |
| 15 |  | 27 | 12 | 1 | 2 |  | 28 | 11 | 3 | 0 |  | 55 | 23 | 4 | 2 |
| 16 |  | 40 | 3 | 2 | 0 |  | 13 | 30 | 0 | 2 |  | 53 | 33 | 2 | 2 |
| 17 |  | 37 | 4 | 4 | 0 |  | 21 | 23 | 0 | 1 |  | 58 | 27 | 4 | 1 |
| 18 |  | 34 | 5 | 3 | 1 |  | 25 | 15 | 0 | 3 |  | 59 | 20 | 3 | 4 |
| 19 |  | 33 | 3 | 0 | 6 |  | 23 | 16 | 3 | 0 |  | 56 | 19 | 3 | 6 |
| 20 |  | 39 | 3 | 2 | 1 |  | 18 | 25 | 1 | 1 |  | 57 | 28 | 3 | 2 |
| 21 |  | 32 | 5 | 6 | 0 |  | 16 | 22 | 0 | 5 |  | 48 | 27 | 6 | 5 |
| 22 |  | 33 | 6 | 3 | 0 |  | 19 | 19 | 0 | 4 |  | 52 | 25 | 3 | 4 |
| **TOT** |  | **713** | **101** | **47** | **33** |  | **424** | **395** | **36** | **39** |  | **1137** | **496** | **83** | **72** |

(b)

|  | **Sensitivity** | **Specificity** | **Accuracy** |
| --- | --- | --- | --- |
| **OFF DETECTION** | 0,68 | 0,96 | 0,91 |
| **ON DETECTION** | 0,92 | 0,92 | 0,92 |
| **GLOBAL VALUES** | 0,86 | 0,94 | 0,91 |

**Figure Captions**

**
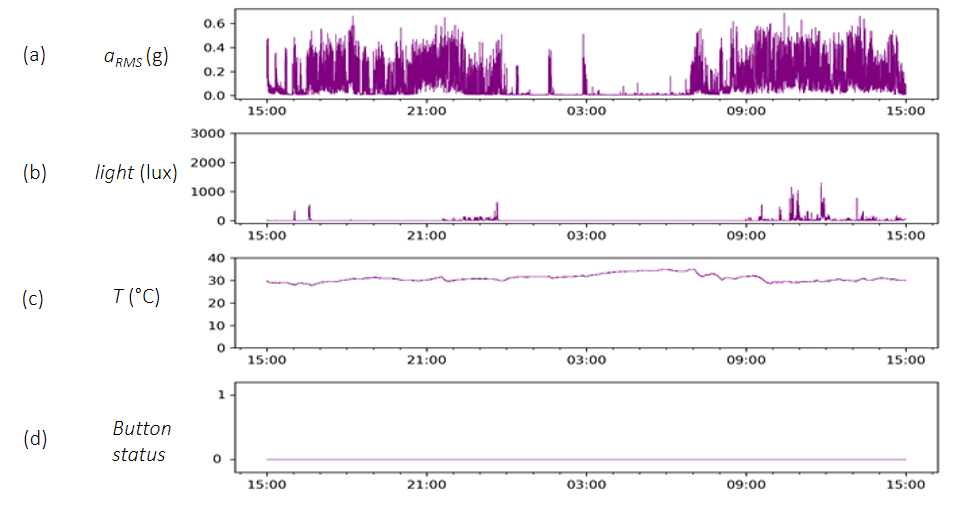
**

**Fig. S1**. Temporal patterns of raw data obtained through the wearable tool for the 24-h acquisition reported in Fig. 1d, including data related to actigraphy 1d (version with sleep analysis is also available – not shown in Figure). (a) RMS acceleration *a_RMS_*. (b) Illuminance, *I*. (c) Near-body temperature, *T*, used to detect not-worn period. (d) Status of event marker button, *S* (0: not press, 1: pressed).

**
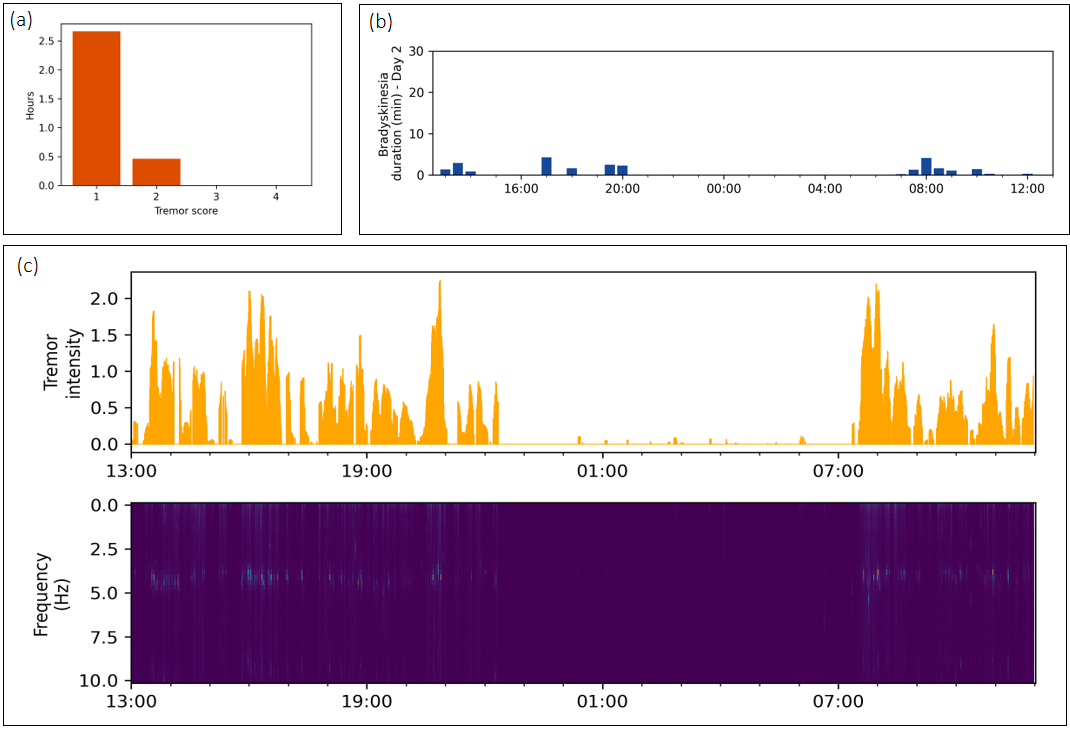
**

**Fig. S2**. Data obtained through the PD-Watch tool for a 72-h acquisition of a PD patient with tremor and bradykinesia and without motor fluctuations. (a) Histogram with the duration tremor for each level of severity expressed in values from 0 to 4, just like the UPDRS “Unified Parkinson's Disease Rating Scale”, for the whole acquisition. (b) Data trend on bradykinesia duration detected by the PD-Watch in 30-min time intervals for a 24-h sequence of the whole 72-h acquisition. (c) Temporal patterns provided by the PD-Watch for a 24-h sequence of the whole 72-h acquisition. First row: the temporal pattern of tremor intensity; second row: a spectrogram of the 24-h recording.


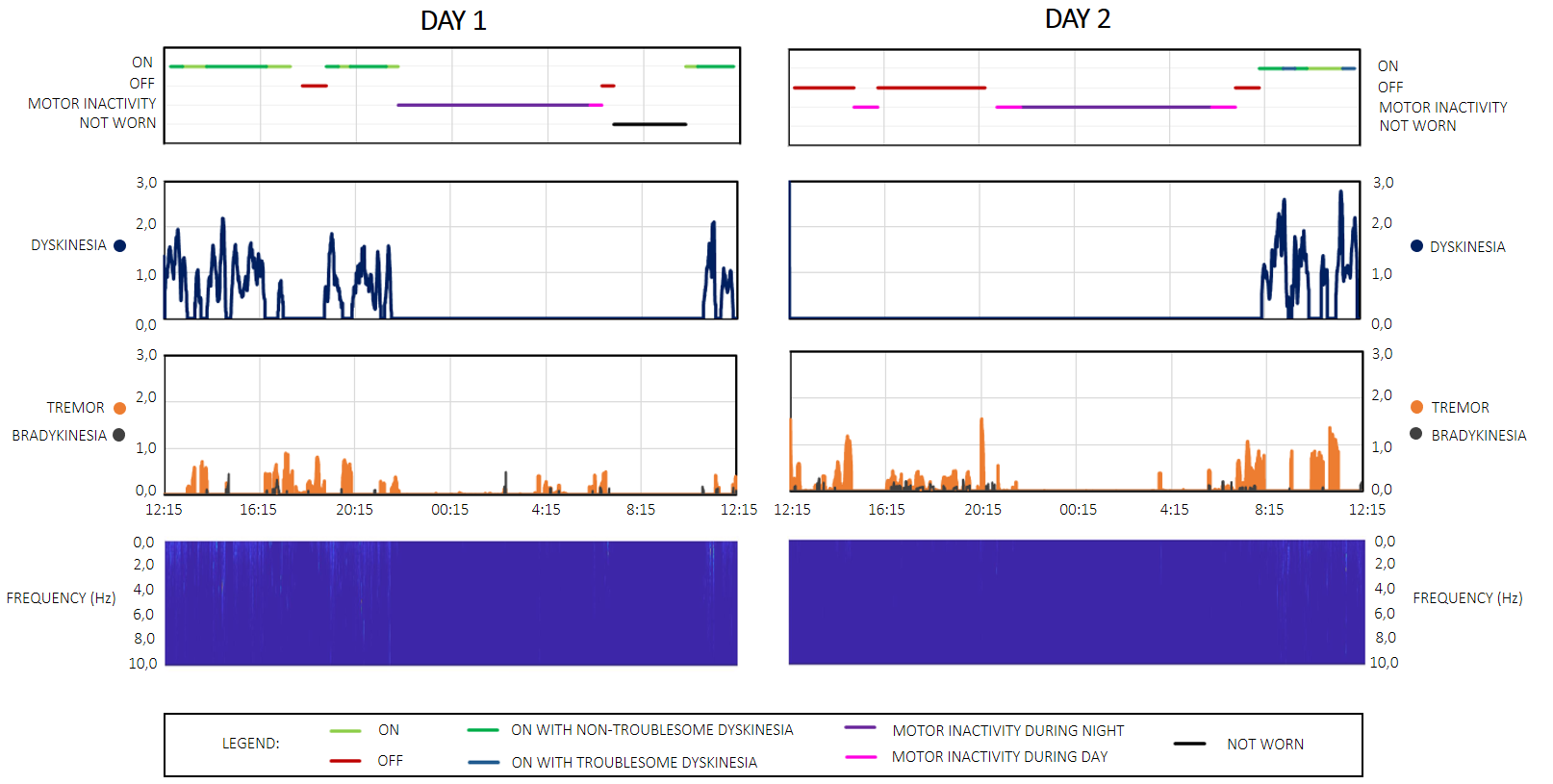


**Fig. S3.** Temporal patterns provided by the PD-Watch for two consecutive days in case of scarce patient adherence to the oral therapy plan. First column: 24-h temporal patterns for the first day; second column: 24-h temporal patterns for the second day. First row: temporal patterns of motor states; second row: temporal pattern of dyskinesia intensity; third row: the temporal pattern of tremor and bradykinesia intensity; fourth row: a spectrogram of the 24-h recording.
